# Responding to maternal, neonatal and child health equipment needs in Kenya: a model for an innovation ecosystem leveraging on collaborations and partnerships

**DOI:** 10.1136/bmjinnov-2019-000391

**Published:** 2020-04-24

**Authors:** Richard Ayah, John Ong'ech, Edwin Maina Mbugua, Rose Chepchumba Kosgei, Katie Waller, David Gathara

**Affiliations:** 1 Science and Technology Park and School of Public Health, University of Nairobi, Nairobi, Kenya; 2 Obstetrics & Gynaecology, Kenyatta National Hospital, Nairobi, Kenya; 3 Concern Worldwide Kenya, Nairobi, Kenya; 4 Obstetrics and Gynaecology, School of Medicine, College of Health Sciences, University of Nairobi, Nairobi, Kenya; 5 Concern Worldwide, New York, New York, USA; 6 Consultant, Nairobi, Kenya

**Keywords:** co-creation, accessible, affordable, innovation

## Abstract

**Background:**

Up to 70% of medical devices in low-income and middle-income countries are partially or completely non-functional, impairing service provision and patient outcomes. In Sub-Saharan Africa, medical devices not designed for local conditions, lack of well-trained biomedical engineers and diverse donated equipment have led to poor maintenance and non-repair. The Maker Project’s aim was to test the effectiveness of an innovative partnership ecosystem network, the ‘Maker Hub’, in reducing gaps in the supply of essential medical devices for maternal, newborn and child health. This paper describes the first phase of the project, the building of the Maker Hub.

**Methods:**

Key activities in setting up the Maker Hub—a collaborative partnership between the University of Nairobi (UoN) and the Kenyatta National Hospital (KNH), catalysed by Concern Worldwide Kenya—are described using a product development partnership approach. Using a health systems approach, a needs assessment identified a medical equipment shortlist. Design thinking with a capacity building component was used by the UoN (innovators, public health specialists, engineers) working closely and with KNH nurses, physicians and biomedical engineers to develop the prototypes.

**Results:**

To date, four medical device prototypes have been developed. Two have been evaluated by the National Bureau of Standards and one has undergone clinical testing.

**Conclusions:**

We have demonstrated an innovative partnership ecosystem that has developed medical devices that have undergone national standards evaluation and clinical testing, a first in Sub-Saharan Africa. Promoting a robust innovation ecosystem for medical equipment requires investment in building trust in the innovation ecosystem.

## Background

The availability, accessibility and effective use of essential medical devices play an important role in the delivery of quality health services. Medicines, vaccines and technologies are among the six building blocks of a health system.^1^ But there has been inadequate funding in the health sector^2^ and poor priority setting practices,^3 4^ leading to low investment in medical products and technologies and resulting in a majority of medical devices used for maternal and child health being donations, which are never enough.^5 6^ It is estimated that up to 70% of medical devices in low-income and middle-income countries (LMICs) are partially or completely non-functional due to various factors; donated devices are often designed in and for high-income settings and are not well suited to low-resource settings, and often arrive without manuals or service contracts.^7^ Furthermore the lack of well-trained biomedical technicians in developing countries to repair the devices when they do inevitably break down exacerbates the problem of unavailability or non-functionality of medical equipment, which has been linked to poor processes of care, impaired service provision and poor patient outcomes.^7–11^ In Kenya, a survey of 22 secondary referral hospitals found that equipment required to undertake a caesarean section were available in 77%–91% (n=22) of the facilities. In terms of types of key medical devices, pulse oximeter and vacuum extractors, which are relatively low-technology devices, were found functional in 3 and 15 out of the 22 surveyed hospitals, respectively.^12 13^ Similar results were observed in a survey of all Nairobi county facilities providing 24 hours 7 days a week newborn care; of the 31 health facilities surveyed, essential equipment that included phototherapy machine, suction machine and warming equipment—radiant heaters, resuscitaire, complete caesarean section sets and diathermy machines—were lacking.^14^

WHO defines medical devices as health technologies that are not medicines, vaccines or clinical procedures used in the prevention, diagnosis or treatment of illness or disease, or for detecting, measuring, restoring, correcting or modifying the structure or function of the body for some health purpose.^15^ Kenya’s Health Policy 2014–2030, the promotion of local production, research and innovations of essential health products and technologies, has been identified as a key action area, where investments will need to be made to facilitate the attainment of set health policy objectives.^16^ Local production using ‘context-aware design’ through product development partnerships (PDPs) is one of the suggested solutions to improving access of medical devices.^17–19^ This is designing devices with flexible technology that fits the needs of the end users in resource-limited settings.^20^ A study including data from 60 resource-poor hospitals located in 11 nations in Africa, Europe, Asia and Central America concluded that a majority of laboratory and medical equipment can be put back into service without importing spare parts as long as the right skills were put to use.^7^ Most developing countries have established institutions with different mandates aimed at helping address local problems and move these countries to developed status. However, while these institutions might have the requisite capacity, they often function in silos.^21^ Therefore, harnessing the strengths of the different institutions through collaborations has been demonstrated as one way of catalysing economic growth and fast-tracking innovation and generating solutions to local problems.^22^ PDPs have been highlighted as one way of strengthening the productive base of the healthcare system through increased local production of medical devices and supplies and reduced dependence on international markets in relation to essential inputs, resulting in improved gross domestic product especially for emerging and developing countries.^22 23^

The Maker Movement for maternal, newborn and child health (MNCH) was established in August 2013 to address gaps in the supply of MNCH medical devices (also referred to as equipment in this manuscript) through a collaborative partnership of key partners in health and academia to create low-cost, high-quality and locally designed and produced essential medical equipment through a network of Makers and MNCH practitioners.^24^ The objective of the Maker Project was to test the potential effectiveness and viability of a network, the ‘Maker Hub’, in reducing gaps in the supply of essential medical devices for MNCH.

The Maker Project was designed to be implemented in two phases. Phase 1, and the focus of this paper, involved building the Maker Hub, conducting a needs assessment of MNCH equipment availability and supply chain bottlenecks which would result in a shortlist of equipment and/or spare parts from which the Maker Hub would prioritise and develop prototypes. The Maker Hub was designed to link local makers (innovators, engineers) and MNCH practitioners including biomedical engineers to design, prototype and test low-cost, high-quality, open-source, locally produced essential devices and spare parts with the objective of improving supply of MNCH equipment to Kenyatta National Hospital (KNH) and its referring health facilities. Phase 2 will involve (1) the production and clinical testing of select MNCH equipment; and (2) the development of business models for the approved equipment and subsequently explore options for commercial production and supply of the equipment.

### Collaborative partnership for an innovation ecosystem

To address gaps in the supply and availability of functional MNCH devices at KNH and lower-level facilities that are responsible for helping women deliver, the ‘Maker Hub’ as a pilot project was set up. The major players in the Maker Hub were KNH and the University of Nairobi (UoN) FabLab, with Concern Worldwide as the catalyst, developing the project design and managing project resources.

KNH, the largest hospital in the region, is a tertiary level, public referral, teaching and research hospital established in 1901.^25^ The hospital has its own procurement/supply chain office and a biomedical engineering facility responsible for maintenance and repair of its equipment. A 2012 government audit at KNH noted that the hospital was unable to repair, maintain or replace equipment in a timely manner.^25^ The report recommended that KNH management developed a management policy to ensure timely acquisition, maintenance and replacement of fixed assets, as well as create a more sustainable financial stream to fund these activities.^25^

The UoN FabLab^26^ was set up in 2011 with the mandate to serve as a rapid prototyping lab within the University Science and Technology Park, which has the university’s mandate to commercialise and incubate research ideas through partnership, innovation and technology transfer. Under the project, a new prototyping lab, christened the ‘Maker Space’, was established and equipped with computer-controlled machining tools that enable the production of prototypes with relative ease, all within an interdisciplinary, sharing environment with an emphasis on hands-on learning using a design thinking approach.

Concern Worldwide Kenya is an international non-governmental humanitarian organisation dedicated to the reduction of suffering and working towards the ultimate elimination of extreme poverty in the world’s poorest countries. Concern Worldwide’s Innovations for MNCH initiative is a multicountry project aimed at identifying and field-testing innovative ways of increasing access to high-impact, low-cost health interventions known to save the lives of women, infants and children in India, Malawi, Sierra Leone, Kenya and Ghana.^27^ The Maker Movement for MNCH was born out of this project.

Project oversight was provided by a committee chaired by the Head of the Division of Reproductive Health in the Ministry of Health (MoH). Additional roles of the MoH included (1) an umbrella institution for policy development, and (2) in the postproject life, the MoH and the county departments for health were the institutions that could allocate government funds for medical devices development and (3) purchase human resource capacity development for implementation and maintenance of medical equipment.

The core team of collaborators that formed the hub in the Maker Movement for MNCH were primarily representatives from KNH and the UoN FabLab, with logistics and monitoring and evaluation support from Concern Worldwide and John Snow Inc.^24^ The KNH team included end users (physicians and nurses from the newborn unit and labour and delivery wards) and biomedical engineers who maintain and repair MNCH equipment. The UoN FabLab team were represented by innovators, public health specialists and engineers. The core team liaised closely with multiple partners and collaborators as part of the implementation strategy. The collaborators were a combination of government, donors and industry that, through being contracted to fabricate prototypes, were kept apprised of project developments, so that they could be part of the broader movement and ensure its sustainability.

**Figure 1:**
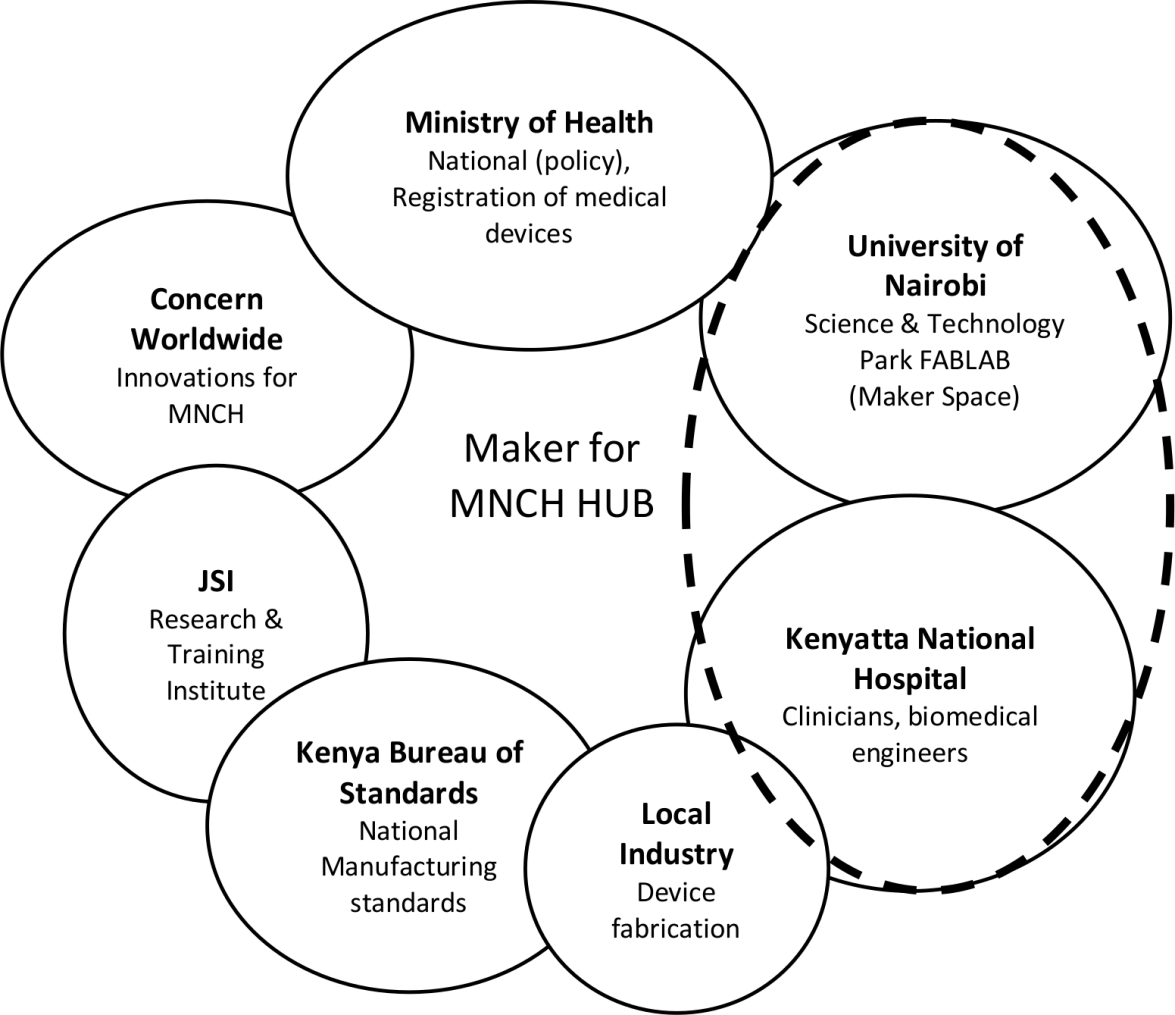
The Maker Hub and collaborators. JSI, John Snow Inc; MNCH, maternal, newborn and child health.

Additional partners that were considered essential for the success of the Maker Project are presented in figure 1. Of note was the role played by the Kenya National Bureau of Standards (KEBS). It provided the Maker Hub with (1) technical guidance on the development of medical devices; (2) conducted relevant quality testing of the medical device prototypes; and (3) training of staff on testing and approval of medical devices designed and built locally.

The Maker Hub pilot forged partnerships between the physicians, nurses and biomedical engineers at KNH and the innovators at the UoN FabLab to build new pieces of equipment or spare parts for select MNCH equipment and improve the supply, availability, reliability and affordability of the equipment.

## Results

The project worked in the labour ward, newborn unit and engineering units. A needs assessment identified the following medical devices and equipment as operating at suboptimal levels or not being available at all due to repeated breakdowns, difficulty in procuring spare parts or incomplete maintenance: (1) patient monitors, (2) resuscitation table, (3) suction machines, (4) drip stands, (5) tables/beds/trolleys, (6) incubators, (7) vacuum extractors, and (8) examination lights. To identify equipment for consideration for innovation, the following criteria for local design and development were used: clinical needs; procurement methods used for primary equipment and spare parts; available infrastructure, what other health system building blocks would be required to support it; health workforce productivity; clinical testing required; business model to be adopted; maker capacity; and incountry manufacturing capacity. Based on these criteria, in June 2014, the Maker Hub prioritised four pieces of equipment (vacuum extractor, phototherapy unit, examination light and suction machine) for design and development. Table 1 provides a summary of equipment operating at suboptimal levels and the inadequacies identified.

**Table 1:** A summary of equipment identified from the needs assessment

	Equipment	Fabrication time (months)	Device classification	Comments (key user concerns)
1	Suction machine	3	Risk class II	Frequent breakdown of available machines. Long readiness turnaround times (sterilisation).
2	Resuscitaire	2–4	Risk class II	The basic baby warmer should have suction capacity as well as oxygen delivery.
3	Infant incubator (whole piece) Spare parts	10 1–3	Risk class II	Many babies put under the few working machines. No way to monitor phototherapy light still within therapeutic dose.
4	Patient monitor (whole piece) Spare parts	6–8	Risk class I (exempt)	Assess probes to determine the required time to build.
5	Examination lights	2	Risk class I (exempt)	Consider solar power as energy source.
6	Vacuum extractor	2	Risk class II	Needs to ensure that the current sample in market does not have intellectual property restrictions.
7	Phototherapy machine	4–8	Risk class II	Many babies put under the few working machines.
8	Oxygen blender	4–7	Risk class II	Centralised system available but subject to procurement constraints.
9	Delivery bed	6	Risk class I (exempt)	Labour ward handles 2–3 times the capacity it was built for.

### Prototyping, design and fabrication process

A human-centred design thinking approach was used, with key components being empathy, fit, buy-in, ownership and uptake.​ The guiding principles of design thinking were introduced to the Maker team over the course of a 3-hour workshop in March 2014 by the Thinkplace Foundation.^28^ The research propositions for design thinking in the Maker applied five components, as outlined in table 2.

**Table 2:** Application of the design thinking to the Maker Project

Design thinking domain	Application of the domain to the Maker Project
Create designer *empathy* for end users.	The designers were the UoN FabLab innovators, and the end users were KNH nurses, physicians and the KNH biomedical team.
*Fit* of problem definition and MNCH intervention with end-user desires, needs and barriers to MNCH care.	For the Maker, this meant the innovators would understand the needs of the KNH clinicians and biomedical teams.
End-user *buy-in* and sense of ownership of the MNCH intervention.	Evidenced through positive perceptions of end users at KNH in the value of the Maker Hub in mitigating the equipment gap at KNH and their willingness to recommend the hub idea as a solution to solving other similar challenges in the health and technology sector.
*Ownership* of the Maker pilot and its outcomes.	Evidenced through perceived/expressed stake of end users at KNH in the success of the Maker, in the value of the hub at solving other similar challenges in the health and technology sector, and in thoughts on the long-term sustainability of the hub.
Demonstrate an increased pace of *uptake* within the Maker.	Uptake seen in the acceptance of end users at KNH of the equipment when prototyped and clinically tested (pace of uptake over time, sustained change over time) and hub members’ acceptance of the concept of the Maker Hub and their interest and stake in keeping it sustainable.

KNH, Kenyatta National Hospital; MNCH, maternal, newborn and child health; UoN, University of Nairobi.

Using the design thinking approach (table 2), the engineers/innovators from the UoN worked closely and consulted frequently with the clinicians (nurses and physicians) and the biomedical engineers of KNH to develop the prototypes. This involved a series of exchange visits to either KNH or FabLab between September 2014 and December 2015 with clinicians and biomedical engineers in KNH and UoN makers, during which the UoN FabLab innovators sought to understand the context within which the clinicians and biomedical staff worked and to enable them to provide feedback on the designs produced by the innovators. In order to understand the concepts of operation, the makers dismantled several machines that were decrepit and non-functional.

The Makers used various software to develop the virtual designs of the medical devices. For mechanical designing, the Maker used SolidWorks, a solid modelling, computer-aided design and engineering program to support the three-dimensional (3D) computer modelling design and development process.^29^ To design electrical circuits, Eagle, an electronic design automation software that enables printed circuit board (PCB) designers to seamlessly connect schematic diagrams, component placement, PCB routing and comprehensive library content,^30^ was used. Once the computer/virtual designs were completed, the Makers fabricated the machines and began creating physical models of the equipment prototypes. This involved dialogue with industry to ensure that designs could be locally fabricated using locally available materials. These fabrications underwent various cumulative changes to take into consideration the feedback received from industry, clinicians and biomedical engineers. The feedback considered the functionality, ease of build and maintenance, user-friendliness, and aesthetics of the machines. The prototypes were reviewed by the clinicians and KNH biomedical engineers to see if the models represented their requests and needs. A second prototype was then evaluated internally (engineering standards) and then by the Kenya Bureau of Standards (using international standards) for public safety and engineering standards. Results of the evaluation were then incorporated into a final prototype approved for clinical testing. From the four short-listed, the suction machine and the phototherapy unit were designed, built and approved by the KEBS. The suction machine successfully underwent clinical trials.^31^ Prototypes for the vacuum and examination light were built, but challenges in procurement of parts from outside the country hampered further development.

### Creating an innovation ecosystem

Creating an enabling environment has been highlighted as a key ingredient in the success of innovations and their commercialisation. While the Maker Hub partners had the human capital and structural capital (includes financial resources, institutional structures/infrastructure) supporting the project, a key part of the project was to create a trusting environment where innovative ideas could be exchanged and implemented in the devices prototyped. Additional activities to create an enabling environment included training, provision of space for the project, equipment and tools, and collaborative meetings with other partners.

## Discussion

The Maker Project sought to test the hypothesis that the ‘Maker Hub’, locally based physicians, nurses and biomedical engineers from KNH, in collaboration with UoN FabLab innovators, can design and build select equipment and spare parts for labour, delivery and newborn care locally. In the initial phase of this work, we have demonstrated that the ‘Maker Hub’ model is a viable model that can address challenges in the social sector through creative collaboration, leadership and governance processes for management. The funding approach used allowed for mechanisms for problem-solving to ensure its long-term sustainability.

While the ‘Maker Hub’ had two major partners working together, the codesign workshops, the inter-institutional meetings and visits, and the partnerships with different stakeholders fostered buy-in at higher levels. We suggest that the collaborative meetings with multiple stakeholders played a key role in the success of the first phase of this project. Thus far, we have developed prototypes for four equipment, with two successfully approved by KEBS and one equipment completed clinical testing. Our findings and other innovation projects implemented in LMIC settings in Haiti and Vietnam to locally develop 3D printing umbilical cord clamps and a firefly phototherapy machine, respectively,^32^ illustrate that it is possible to locally produce medical devices when appropriate systems are put in place.

Our experience in setting up an innovation ecosystem resonates with PDP approaches used by industries to help codevelop medical devices and vaccines, which have been shown to be effective.^33 34^ The collaborative nature of the project ensured that several determinants of innovation were addressed.^34^ The three primary institutions involved all have a mandate to serve the public which together with oversight from MoH made implementation of intellectual property rights relatively easy and non-contentious. The innovations to the medical devices were registered by the UoN intellectual property rights office, acknowledging individual contributions of innovators, while the final products are owned by the project.

Second, involvement of KEBS ensured that the design and the devices built were safe and up to international standards. Regulatory framework is an essential component of local device production, yet while pharmaceutical products are widely regulated in the region, regulatory capacity is limited, with only South Africa in Sub-Saharan Africa having a regulatory framework recognising medical devices in their own category.^35 36^ This project is, we believe, the first in the region to design, build and clinically test medical devices. The project therefore spent considerable time engaging and supporting the Kenya Bureau of Standards in drafting standards and requisite documentation needed to test the medical devices. While this was a tremendous responsibility that the project undertook willingly and successfully, it diverted attention and resources. The lack of a clear policy and regulatory framework is a considerable barrier to innovation and local production of medical devices.^35^

A third consideration, in addition to building the capacity of the biomedical staff, was indirect assessment of local industry capacity to build and maintain parts for the devices, critical in building resilience and sustainability of the health system.^37^ Evidence shows that some locally produced simple devices can be more affordable than foreign imports, often due to reduced costs of locally available materials, transport, improved supply chain and low maintenance costs due to being locally available after market support.^38^ An additional benefit is that local production is closely linked with innovation through development of novel solutions to address identified needs, while adopting knowledge and fabricating skills from industry.^39^

The team-based capacity building (users, biomedical and UoN FabLab makers) approach used in the Maker Hub project has opened up opportunities for other areas of collaboration. For example, the Maker Space has now partnered with UNICEF and Philips Foundation to develop medical devices for LMICs and has been recognised by the FabLab community as a centre for MNCH innovation in Africa. However, there were challenges, and among them the high attrition rate of makers (mainly engineering and medical students), who were essentially volunteers, who dropped out when their academic programmes became too taxing. The lack of locally available high-quality materials and equipment for fabrication led to redesign delays and necessitated international procurement, which came with its own set of challenges especially in procurement delays and bureaucracies associated with buying small quantities of one-off components, leading to two devices not being built in time for testing.

In conclusion, in this first phase of the project, we have demonstrated the capacity to locally design build and clinically test essential medical devices and equipment. We have described the process of building a collaborative team across the university, hospital, industry and government, catalysed by a non-governmental organisation. We draw attention to the considerable effort needed to fostering innovation by first investing in building trust among the institutions within the innovation ecosystem. To promote a robust innovation ecosystem to design and build low-cost, contextually appropriate medical equipment, governments in LMICs and funding agencies should increase financing and opportunities that support collaboration among local research, medical practice and regulatory institutions. The next step for the medical devices built in this project is to establish the financing and deployment of sufficient numbers of the medical devices and equipment to impact on patient health.
